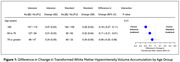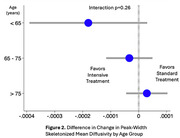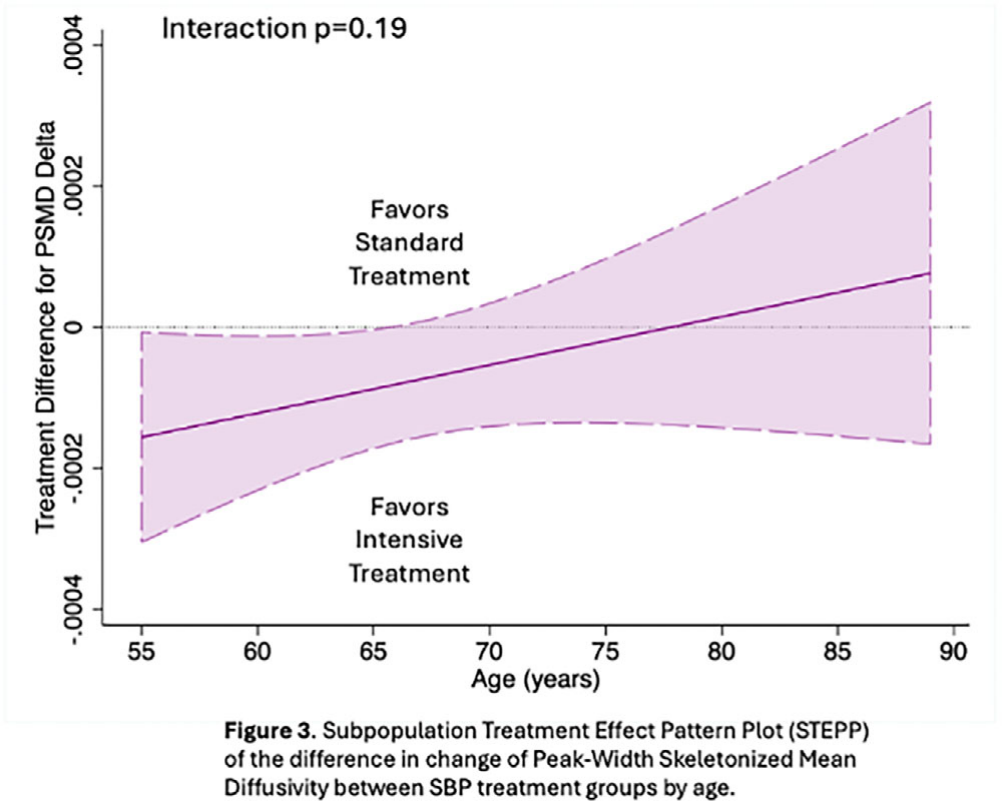# Potential Relative Effect of Intensive Systolic Blood Pressure Control on Preventing White Matter Damage Across Age Groups

**DOI:** 10.1002/alz.094121

**Published:** 2025-01-09

**Authors:** Jeremy J. Pruzin, Kyle C Kern, David M. Reboussin, William C. Cushman, Aditi Gupta, Clinton B. Wright, Jeff D. Williamson, Pierre N. Tariot, Ilya M. Nasrallah, Nicholas M. Pajewski

**Affiliations:** ^1^ Banner Alzheimer's Institute, Phoenix, AZ USA; ^2^ UCLA David Geffen School of Medicine, Los Angeles, CA USA; ^3^ Wake Forest University School of Medicine, Winston‐Salem, NC USA; ^4^ University of Tennessee Health Science Center, Memphis, TN USA; ^5^ University of Kansas Medical Center, Kansas City, KS USA; ^6^ NINDS, Bethesda, MD USA; ^7^ Department of Radiology, University of Pennsylvania, Philadelphia, PA USA

## Abstract

**Background:**

In the Systolic Blood Pressure Intervention Trial (SPRINT), intensive systolic blood pressure (SBP) lowering slowed progression of white matter injury (WMI) on MRI. We hypothesized that intensive lowering would be equally as effective and may confer greater benefits for brain health at younger ages compared to older ages. We tested whether the relative effects of intensive lowering on WMI differed by age using 2 MRI measures: white matter hyperintensity volume (WMHv) and peak‐width skeletonized mean diffusivity (PSMD) in SPRINT.

**Method:**

Participants were age =50 with cardiovascular risk and randomized to intensive (SBP goal <120 mm Hg) or standard treatment (SBP goal <140 mm Hg). We calculated WMHv and PSMD in a subgroup of participants who had a baseline and follow‐up MRI. WMHv were inverse‐hyperbolic sine‐transformed, and the ratio of follow‐up to baseline used to quantify progression. PSMD progression was quantified as a difference. Using mixed‐effects linear models, we estimated the effects of age on relative progression in MRI markers between groups. Age effects were investigated as categorical (<65, 65‐75, or =75 years) or continuous.

**Result:**

For participants in the intensive group (n=251 with follow‐up), mean SBP was 122 mmHg versus 135 mmHg in the standard group (n=201) over a median 3.9‐year MRI interval. The largest treatment effect on WMHv progression was found in the <65 age group (i.e. greatest relative reduction with intensive treatment): ‐0.19, 95%CI [‐0.27, ‐0.11]), followed by the 65‐75 age group (‐0.11, [‐0.21, ‐0.01]), and least in the >75 age group (‐0.06, [‐0.20, ‐0.08]) (Figure 1). Intensive treatment resulted in a 73%, 52%, and 20% reduction in WMHv progression respectively, although this was not statistically significant (p=0.21). Analyses of PSMD progression produced similar results (Figure 2). When using age as a continuous measure, the beneficial effect of intensive treatment waned with increasing age (Figure 3) without reaching statistical significance (p=0.19).

**Conclusion:**

Intensive SBP lowering was equally as effective and may have a greater relative effect on reducing WMI when implemented at younger ages compared to older ages. Large prospective interventional studies that include younger individuals are needed to determine the effects of SBP on white matter integrity across the lifespan.